# *Eucommia ulmoides Oliver*-*Tribulus terrestris* L. Drug Pair Regulates Ferroptosis by Mediating the Neurovascular-Related Ligand-Receptor Interaction Pathway- A Potential Drug Pair for Treatment Hypertension and Prevention Ischemic Stroke

**DOI:** 10.3389/fneur.2022.833922

**Published:** 2022-03-08

**Authors:** Qian Zhang, Jie Yang, Chuanhua Yang, Xuesong Yang, Yongzhi Chen

**Affiliations:** ^1^Department of Science and Technology Office, The Affiliated Hospital of Shandong University of Traditional Chinese Medicine, Jinan, China; ^2^Department of Cardiology, The Affiliated Hospital of Shandong University of Traditional Chinese Medicine, Jinan, China; ^3^Department of Vascular Surgery, The Affiliated Hospital of Shandong University of Traditional Chinese Medicine, Jinan, China

**Keywords:** stroke, hypertension, *Eucommia ulmoides Oliver*, *Tribulus terrestris* L, ferroptosis, network pharmacology Taubert D

## Abstract

**Background:**

In this study, we used the network pharmacology approach to explore the potential disease targets of the *Eucommia ulmoides Oliver* (EUO)-*Tribulus terrestris* L. (TT) drug pair in the treatment of hypertension-associated neurovascular lesions and IS via the ferroptosis pathway.

**Methods:**

We used the Traditional Chinese Medicine Systems Pharmacology Database and Analysis Platform to search for the key active compounds and targets of the drug pair. Based on the GeneCards database, the relevant targets for the drug pair were obtained. Then, we performed the molecular docking of the screened core active ingredients and proteins using the DAVID database and the R AutoDock Vina software. Based on the GSE22255 dataset, these screened target proteins were used to build random forest (RF) and support vector machine (SVM) models. Finally, a new IS nomogram prediction model was constructed and evaluated.

**Results:**

There were 36 active compounds in the EUO-TT drug pair. CHRM1, NR3C1, ADRB2, and OPRD1 proteins of the neuroactive ligand-receptor interaction pathway interacted with the proteins related to the ferroptosis pathway. Molecular docking experiments identified 12 active ingredients of the drug pair that may tightly bind to those target proteins. We constructed a visual IS nomogram prediction model using four genes (CHRM1, NR3C1, ADRB2, and OPRD1). The calibration curve, DCA, and clinical impact curves all indicated that the nomogram model is clinically applicable and diagnostically capable. CHRM1, NR3C1, ADRB2, and OPRD1, the target genes of the four effective components of the EUO-TT drug pair, were considered as risk markers for IS.

**Conclusions:**

The active ingredients of EUO-TT drug pair may act on proteins associated with the neuroactive ligand-receptor interaction pathway to regulate ferroptosis in vascular neurons cells, ultimately affecting the onset and progression of hypertension.

## Introduction

Ischemic stroke (IS), one of the neurovascular disorders, is a common complication of hypertension, the second most common cause of death worldwide, and has become a major public health problem. Stroke affects approximately 15 million people worldwide each year, resulting in 5 million deaths and 5 million disabilities (1). A study based on the Global Burden of Disease, Injuries and Risk Factors (GDB2010) showed that stroke mortality has increased by 26% since 1990 (2). A prospective observational study of 61 populations worldwide (~1 million people aged 40–89 years) found a positive association between stroke risk and blood pressure levels (3). The earliest appearances are changes in arterial elastic function and an increase in pressure wave conduction velocity. The continuous increase in blood pressure progressively damages the integrity of the vascular endothelium resulting in structural changes to the vessel wall. The higher the blood pressure level, the more severe the damage. There is a strong causal relationship between blood pressure levels and stroke morbidity and mortality. Although the effect of western medicine in the treatment of hypertension is precise, it is mainly based on the control of blood pressure. This treatment regimen has several disadvantages, such as high dependence on medicines, numerous side effects, and heavy economic burden due to long-term use. Chinese medicine has unique advantages in the prevention and treatment of hypertension and neurovascular related diseases since they target multiple pathways and are less toxic.

EUO is a living fossil plant. Recent studies have found more than a hundred compounds that can be extracted from EUO. Monomeric compounds and extracts of EUO have a wide range of pharmacological effects in the treatment of several diseases, including hypertension, hyperlipidemia, diabetes, and osteoporosis (4, 5). The active ingredients of EUO that are anti-hypertensive are divided into four major groups: lignans, phenylpropanoids, flavonoids, and cyclic enol ether terpenes. Among these, lignans are the most studied class of chemical compounds which may regulate nitric oxide levels, renin-angiotensin system, and direct arterial diastole (6). The lignans in EUO exert anti-hypertensive effects in spontaneously hypertensive rats (SHR), presumably by inhibiting aldose reductase (AR), inhibiting the Ang II-induced inflammation and oxidative stress signaling pathways, and preventing cardiovascular remodeling (7, 8). Moreover, lignans can protect against renal damage caused by hypertension (9). Other components of EUO, like phenylpropanoid caffeic acid, lowers the blood pressure by inducing nitric oxide synthase and promoting nitric oxide synthesis (10, 11). Further, quercetin, a flavonoid, has the effect of endothelium-dependent vasodilatation (11, 12). Thus, EUO contains a variety of anti-hypertensive components that may protect against hypertension-induced ischemic stroke.

Another Chinese medicine, TT, has been shown by modern pharmacological studies to be anti-hypertensive, diuretic, hypolipidemic, and anti-atherosclerotic. It also reduces left ventricular remodeling and improves cardiac function in the early post-myocardial infarction period (13–15). Phillips et al. studied the effects of the methanolic and aqueous extracts of TT on blood pressure and perfused mesenteric vascular bed in rats, and showed that TT resulted in a dose-related reduction in blood pressure of SHR (13). Further, Sharifi et al. found that TT lowers the blood pressure in hypertensive rat models by inhibiting the angiotensin-converting enzyme (16). Active components isolated from TT also exert anti-inflammatory effects by inhibiting inflammatory mediators and cytokines, including IL-6, IL-10, and TNF-α (17). Inflammation is one of the several pathogeneses of hypertension, and inflammatory factors such as IL-6 are involved in the development and progression of hypertension (18–20). Thus, the components of Tribulus terrestris L have a potential key role in the prevention and treatment of hypertension through several pathways.

Recent studies have found that treatment of ferroptosis may be an important means of preventing and improving the prognosis of patients with IS (21, 22). Ferroptosis is a novel form of cell death discovered by Dolma et al. (23). The main feature of ferroptosis is the iron ion-dependent accumulation of large amounts of lipid peroxides during cell death (24). P53, System Xc-, glutathione peroxidase 4 (GPX4), and Fe are the core components of the ferroptosis pathway. Moreover, ferroptosis-related genes are mainly involved in the oxidative stress response (25). Oxidative stress and associated oxidative damage may be the main cause of vascular injury and may be involved in hypertension, where increased utilization and/or decreased inactivation of reactive oxygen species (ROS) is key to vasoconstriction dysfunction (26–28). GPX4 is a highly active antioxidant enzyme in the basal body that scavenges ROS and prevents oxidative stress. Hence, its decline, either by using a GPX4 inhibitor or knockdown of the GPX4 gene, is associated with the development of pre-eclampsia and a significant increase in intracellular ROS and lipid peroxidation, thereby inducing ferroptosis (29–31). Recent research has established a clear correlation between ferroptosis and hypertensive brain injury, indicating that increased blood pressure results in a large drop in GPX4 and GSH levels in brain tissue, resulting in iron overload. Excessive iron levels in the brain enhance oxidative stress and lipid peroxidation, finally resulting in brain damage (32). Further, hypertensive patients have decreased activity and expression of GPX (33). Inhibition of endothelial ferroptosis may help prevent endothelial cell apoptosis and calcification due to hypertension (34, 35). Thus, ferroptosis-related pathways may play a role in hypertension related vascular endothelial damage, but there is a lack of direct research to prove this.

In previous study, the ferroptosis of neuronal cells in hypertension is important for neurovascular diseases progression (22). This may be a critical mechanism for neurological mediation of the progression of hypertension. According to traditional Chinese medicine theory, the EUO-TT medication combination may help increase cerebrovascular health in hypertension patients, however further research is needed to confirm this idea. We hypothesize that the EUO-TT drug pair may aid in inhibiting ferroptosis in the hypertensive milieu, hence reducing hypertensive damage to the brain's endothelial cells. Treatment with the EUO-TT medication combination may help prevent and improve the prognosis of people with IS.

In this study, we used a network pharmacology approach to construct a “compound-target-disease” network through bioinformatics analysis to unravel the pharmacological basis and mechanism of action of the drug pair of EUO-TT in the treatment of hypertension-associated neurovascular lesions through the ferroptosis pathway. We believe that the findings of our study will provide a pharmacological basis for the clinical treatment of hypertension using Chinese medicine.

## Materials and Methods

### Acquisition of Effective Compounds and Action Targets of EUO-TT

In this study, we searched for the chemical composition of EUO and TT through the pharmacology platform of the Chinese medicine network. We extracted the main active compounds of each medicine with the screening conditions of oral bioavailability ≥30% and drug-likeness ≥0.18 (36).

### Prediction of Hypertension Disease Target

The hypertension-related targets were searched by MOL ID numbers (expressed as protein name) of compounds using the Traditional Chinese Medicine Systems Pharmacology Database and Analysis Platform (TCMSP). Further, the gene names corresponding to each target were searched by the Uniport database (37). Based on the GeneCards database, the relevant genes were searched by the keyword “hypertension” and intersected with the key active compounds of the pathway to obtain the relevant targets of the EUO-TT drug pair for the treatment of hypertension (38).

### Protein-Protein Interaction (PPI) Network Construction and Key Target Screening

After importing the intersecting targets into the STRING database and restricting the study species to humans, we obtained the PPI network of EUO-TT drug pair acting on neuroactive ligand-receptor interaction pathway and ferroptosis pathway (39). The topological analysis of the PPI network was performed with the help of “Network Analyzer” in Cytoscape 3.7.1 software. The targets with degrees greater than the average of the degree of freedom, betweenness, and closeness were selected as key targets of the neuroactive ligand-receptor interaction pathway and ferroptosis pathway (39).

### Construction and Analysis of the Neuroactive Ligand-Receptor Interaction Pathway-Ferroptosis Pathway Action Target Network

The active compounds and their targets in the neuroactive ligand-receptor interaction pathway were imported into Cytoscape 3.7.1 software, and the “STRING database” function was used to analyze the neuroactive ligand-receptor interaction pathway-ferroptosis pathway target network. The key compounds of EUO-Tribulus terrestris L drug pair mediating neuroactive ligand-receptor interaction pathway to coordinate ferroptosis pathway for the treatment of hypertension were screened based on compound-target connectivity.

### Gene Ontology (GO) Functional Enrichment Analysis and Kyoto Encyclopedia of Genes and Genomes (KEGG) Pathway Analysis

In order to further understand the core target gene functions and the main pathways of action of EUO-Tribulus terrestris L in the treatment of hypertension, the drug-disease related targets were entered into the DAVID database (40). The species “homo sapiens” was selected, and a threshold value of adjust *p* < 0.05 was set for GO function enrichment and KEGG pathway analysis, of which results were visualized by R programming software (41, 42).

### Molecular Docking

As described in previous studies, we molecularly docked the screened core active ingredient to the core protein to verify the interaction strength (43). The active ingredient structures were obtained from the PubChem database and the protein structures were obtained from the Protein Data Bank (PDB) (44, 45). The 3D structures of the active ingredients (mol2 format) were downloaded, and operations such as hydrogenation, charge addition, detection of ligand roots, and search and definition of rotatable bonds were performed with AutoDock. Molecular docking was performed using AutoDock Vina software, and the results were optimized for mapping and further analysis with the help of Pymol software (46, 47). R software was used to analyze and thermogram small molecule bound amino acids.

### Data Acquisition and Pre-processing

The GSE22255 dataset is an ischaemic stroke-related microarray dataset obtained from the GEO database (48). The dataset consisted of 20 IS patients and 20 age-matched controls. Analysis of variance was done using the “limma” package of R software (version 4.0.5) and the remove Barch effect function was used to remove batch effects. Gene expression was used for normalization analysis and *p*-values less than 0.05 were defined as significant differences.

### Building Predictive Models

In short, the predictive model was constructed with reference to previous studies (49–53). With CHRM1, NR3C1, ADRB2, and OPRD1 as dependent variables and the presence or absence of IS as the dependent variable, the “pacman” package of R software was used to build random forest (RF) and support vector machine (SVM) models. A receiver operating characteristic (ROC) curve, reverse cumulative distribution of |residual|, and boxplots of |residual| were plotted to select the most appropriate model. Then, using the “mlbench” and “caret” programmes, the cross curve was plotted to identify the relevance of each element. Following that, the “rms” function was used to build the nomogram prediction model. Additionally, calibration curves, DCA curves, and clinical effect curves were developed to validate the model's predictive ability (54, 55).

## Results

### Extraction of Effective Compounds and Action Targets of EUO-TT Drug Pair

The TCMSP analysis platform was used to search the main active ingredients of the EUO-TT drug pair. The active ingredients of each drug were screened and the targets of each compound were obtained according to the active ingredient “MOL ID”. Among them, 25 active compounds were screened from EUO, 12 from TT, and one duplicate compound from both EUO and TT (MOL000422: kaempferol). We found 36 active compounds after de-weighting. Further, 30 compounds were found to correspond to 114 genes, and the gene names of each target were obtained from Uniprot (Table 1).

**Table 1:** Detailed information on active compounds from *Eucommia ulmoides Oliver* and *Tribulus terrestris* L.

**Drug**	**MOL ID**	**Molecule name**	**OB**	**Drug-likeness**
*Eucommia ulmoides Oliver*	MOL000073	ent-Epicatechin	48.96	0.24
*Eucommia ulmoides Oliver*	MOL000098	quercetin	46.43	0.28
*Eucommia ulmoides Oliver*	MOL000211	Mairin	55.38	0.78
*Eucommia ulmoides Oliver*	MOL000358	beta-sitosterol	36.91	0.75
*Eucommia ulmoides Oliver*	MOL000422	kaempferol	41.88	0.24
*Eucommia ulmoides Oliver*	MOL000443	Erythraline	49.18	0.55
*Eucommia ulmoides Oliver*	MOL002058	40957-99-1	57.20	0.62
*Eucommia ulmoides Oliver*	MOL004367	olivil	62.23	0.41
*Eucommia ulmoides Oliver*	MOL005922	Acanthoside B	43.35	0.77
*Eucommia ulmoides Oliver*	MOL006709	AIDS214634	92.43	0.55
*Eucommia ulmoides Oliver*	MOL007059	3-beta-Hydroxymethyllenetanshiquinone	32.16	0.41
*Eucommia ulmoides Oliver*	MOL007563	Yangambin	57.53	0.81
*Eucommia ulmoides Oliver*	MOL009007	Eucommin A	30.51	0.85
*Eucommia ulmoides Oliver*	MOL009009	(+)-medioresinol	87.19	0.62
*Eucommia ulmoides Oliver*	MOL009015	(–)-Tabernemontanine	58.67	0.61
*Eucommia ulmoides Oliver*	MOL009027	Cyclopamine	55.42	0.82
*Eucommia ulmoides Oliver*	MOL009029	Dehydrodiconiferyl alcohol 4,gamma'-di-O-beta-D-glucopyanoside_qt	51.44	0.40
*Eucommia ulmoides Oliver*	MOL009030	Dehydrodieugenol	30.10	0.24
*Eucommia ulmoides Oliver*	MOL009031	Epiquinidine	68.22	0.40
*Eucommia ulmoides Oliver*	MOL009038	GBGB	45.58	0.83
*Eucommia ulmoides Oliver*	MOL009042	Helenalin	77.01	0.19
*Eucommia ulmoides Oliver*	MOL009047	(+)-Eudesmin	33.29	0.62
*Eucommia ulmoides Oliver*	MOL009053	4-[(2S,3R)-5-[(E)-3-hydroxyprop-1-enyl]-7-methoxy-3-methylol-2,3-dihydrobenzofuran-2-yl]-2-methoxy-phenol	50.76	0.39
*Eucommia ulmoides Oliver*	MOL009055	hirsutin_qt	49.81	0.37
*Eucommia ulmoides Oliver*	MOL009057	liriodendrin_qt	53.14	0.80
*Tribulus terrestris* L.	MOL000354	isorhamnetin	49.60	0.31
*Tribulus terrestris* L.	MOL000359	sitosterol	36.91	0.75
*Tribulus terrestris* L.	MOL000422	kaempferol	41.88	0.24
*Tribulus terrestris* L.	MOL000483	Moupinamide	118.35	0.26
*Tribulus terrestris* L.	MOL008559	(2aR,2'R,4R,6aR,6bS,8aS,8bR,11aS,12aR,12bR)-4-((S)-2-(2,6-dimethylphenyl)propoxy)-5',5',6a,8a-tetramethyl-8-methylenedocosahydro-1H-spiro[pentaleno[2,1-a]phenanthrene-10,2'-pyran]	59.49	0.28
*Tribulus terrestris* L.	MOL008563	(3R,8S,9S,10R,13R,14R,17S)-17-((2S,5R)-5-ethyl-6-methylheptan-2-yl)-3-hydroxy-10,13-dimethyl-3,4,8,9,10,11,12,13,14,15,16,17-dodecahydro-1H-cyclopenta[a]phenanthren-7(2H)-one	40.93	0.79
*Tribulus terrestris* L.	MOL008567	(3R,7R,8S,9S,10S,13R,14S,17R)-17-((2R,5S)-5-ethyl-6-methylheptan-2-yl)-3,10-dimethyl-2,3,4,7,8,9,10,11,12,13,14,15,16,17-tetradecahydro-1H-cyclopenta[a]phenanthren-7-ol	34.21	0.76
*Tribulus terrestris* L.	MOL008568	(Z)-3-(3,4-dihydroxyphenyl)-N-[2-(4-hydroxyphenyl)ethyl]acrylamide	113.25	0.24
*Tribulus terrestris* L.	MOL008569	β-sitosterol-β-D-glucopyranoside	32.41	0.71
*Tribulus terrestris* L.	MOL008588	terrestriamide	114.09	0.29
*Tribulus terrestris* L.	MOL008590	(2aR,2'S,4R,4'R,5'S,6aS,6bS,8aS,8bR,9S,11aR,12aR,12bR)-4,4'-dihydroxy-5',6a,8a,9-tetramethylicosahydro-1H-spiro[pentaleno[2,1-a]phenanthrene-10,2'-pyran]-8(2H)-one	58.74	0.76
*Tribulus terrestris* L.	MOL008593	(2aR,5S,6aS,6bS,8aS,8bS,11aS,12aR,12bR)-10-isopentyl-6a,8a,9-trimethyl-2,2a,3,4,5,6,6a,6b,7,8,8a,8b,11a,12,12a,12b-hexadecahydro-1H-naphtho[2',1':4,5]indeno[2,1-b]furan-5-ol	39.21	0.84

### Enrichment Analysis of the Targets of Active Compounds in the EUO-TT Drug Pair

**Figure 1:**
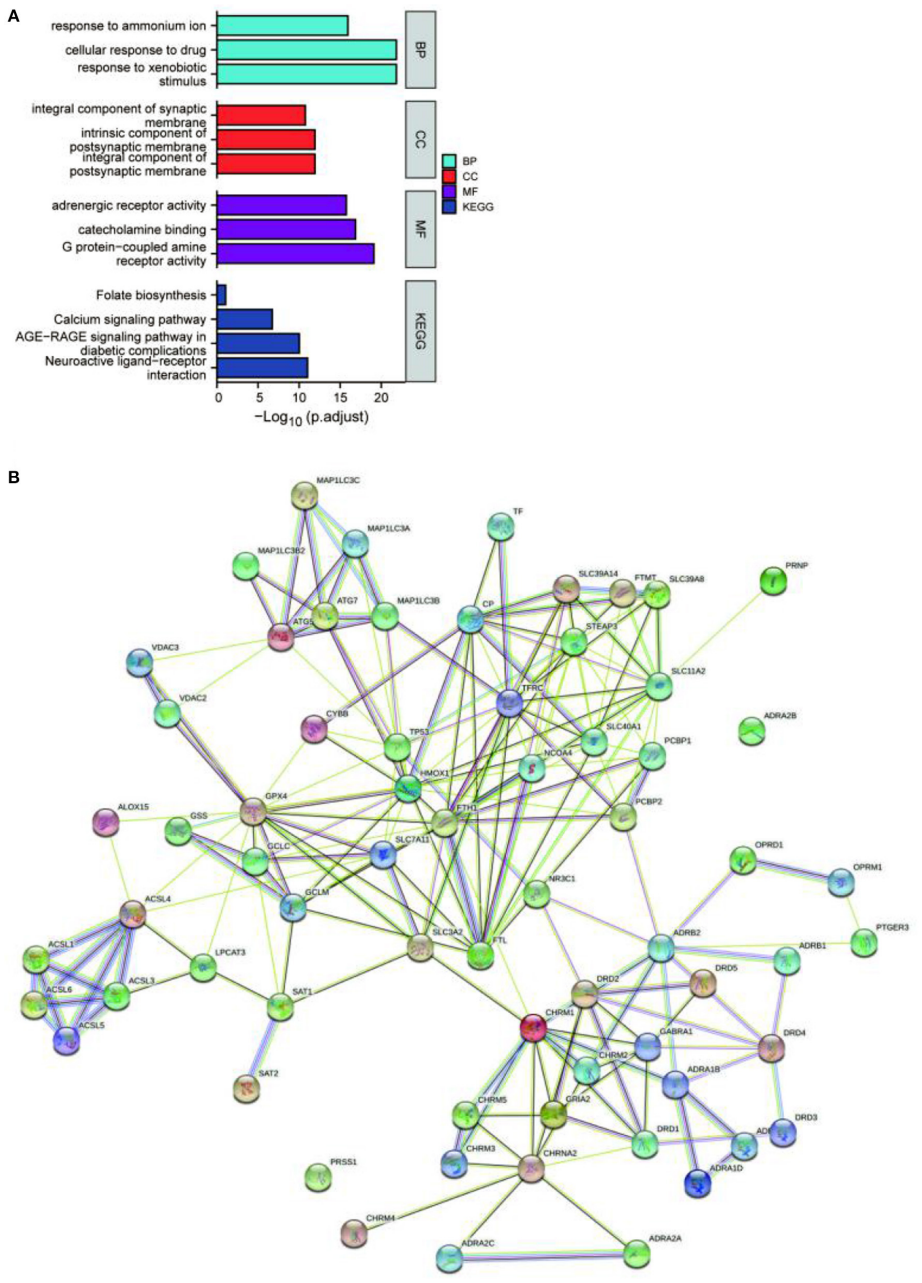
Enrichment analysis and protein-protein interaction (PPI) network of the active compound targets of EUO-TT L. GO functional analysis and KEGG enrichment analysis of the active compound targets of EUO-TT L. drug pair **(A)**; PPI network analysis of the target genes of the neuroactive ligand-receptor interaction pathway, and the ferroptosis pathway mediated by the drug pair **(B)**.

The potential target genes of the drugs were imported into the DAVID 6.8 database, the list of target genes was entered, the species was limited to “Homo sapiens”, and the threshold was set to *P* < 0.05 for GO functional analysis and KEGG pathway enrichment analysis. The pathways and functional information with the top P-values in each type of process and pathways were selected, where smaller *P*-values and longer bar lengths indicated the strongest enrichment significance (Figure 1A). The top-ranked biological processes (BP), cellular components (CC), and molecular functions (MF) included: response to ammonium ion, cellular response to drug, response to xenobiotic stimulus, integral component of synaptic membrane, intrinsic component of postsynaptic membrane, integral component of postsynaptic membrane, adrenergic receptor activity, catecholamine binding, and G protein-coupled amine receptor activity. The top-ranked KEGG included: Folate biosynthesis, Calcium signaling pathway, AGE-RAGE signaling pathway in diabetic complications, and Neuroactive ligand-receptor interaction. Among them, the target genes of the EUO-TT drug pair were found to be significantly enriched in the neuroactive ligand-receptor interaction pathway. Therefore, we speculated that this drug pair may regulate the ferroptosis pathway by modulating the neuroactive ligand-receptor interaction pathway.

### Potential Mechanism of Neuroactive Ligand-Receptor Interaction Pathway Mediated by the Active Compounds of EUO-TT Drug Pair for the Regulation of Ferroptosis

**Figure 2:**
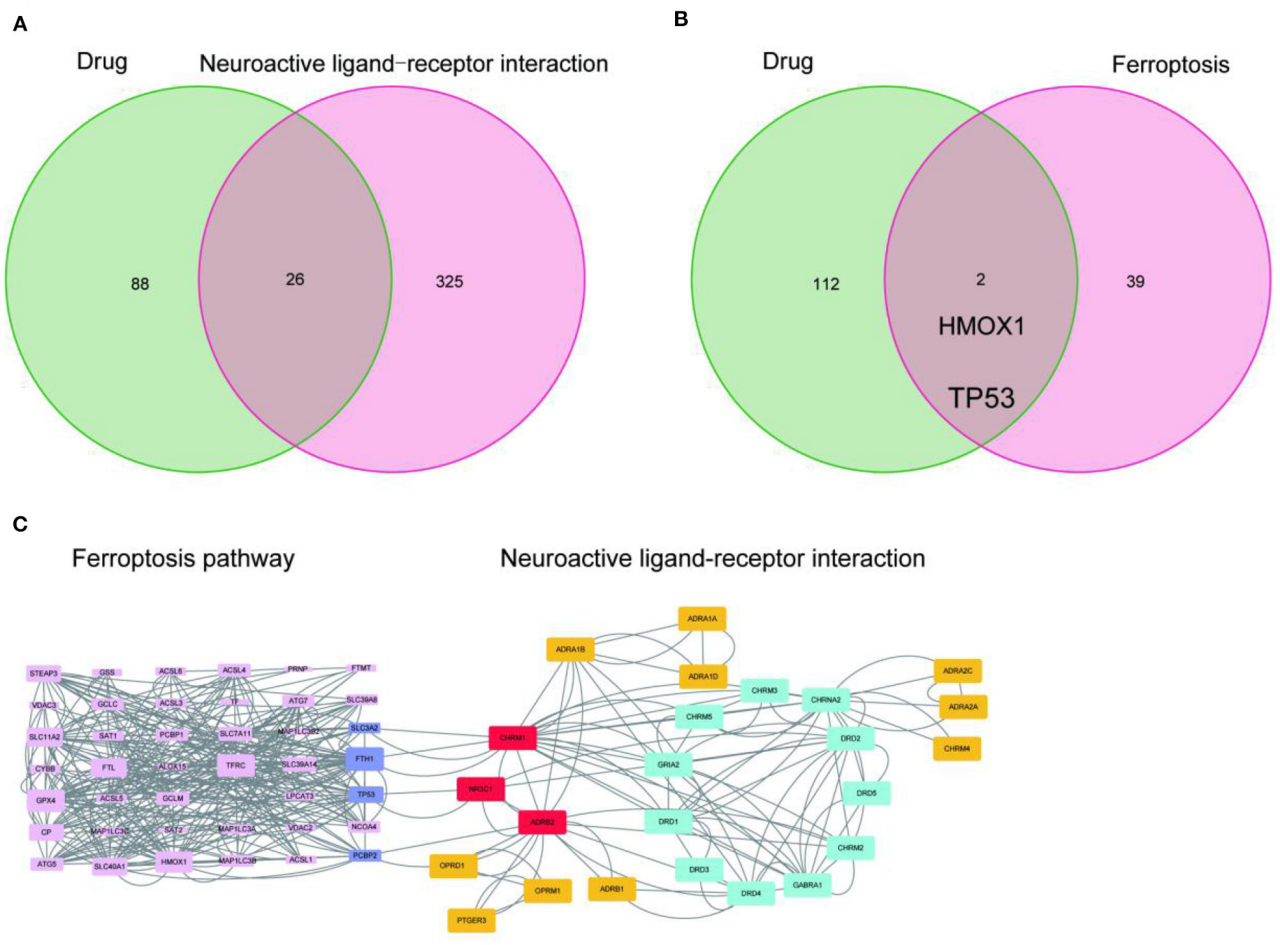
Network analysis of the active compounds of EUO-TT L. mediating the neuroactive ligand-receptor interaction pathway regulating ferroptosis. Intersection analysis of drug-neuroactive ligand-receptor interaction pathway **(A)**; Intersection analysis of drug-ferroptosis pathway **(B)**; PPI network of neuroactive ligand-receptor interaction pathway - ferroptosis pathway **(C)**.

Since the target genes of the drug pair were significantly enriched in the neuroactive ligand-receptor interaction pathway, we completed the intersection analysis of the drug-neuroactive ligand-receptor interaction pathway, and 26 intersection targets were identified (Figure 2A). However, the drug-ferroptosis intersection analysis identified only two intersection targets (Figure 2B). We, therefore, hypothesized that the neuroactive ligand-receptor interaction pathway may be the direct targeting pathway of EUO-TT drug pair, while the ferroptosis pathway may be the indirect pathway. The STRING database was used for the PPI analysis of the 26 intersecting targets of the drug-neuroactive ligand-receptor interaction pathway and ferroptosis pathway proteins (from the KEGG database). The study species was set as “Homo sapiens” to obtain the PPI network of the drug pair (Figure 1B). We used the default parameters on the web page, and the file was saved in TSV format and imported into Cytoscape software for topological analysis of the PPI network. The nodes in Figure 1B were selected out in Figure 2C. Combined with the information on the degrees of freedom, degree, and centrality, the neuroactive ligand-receptor interaction pathway - ferroptosis pathway regulatory network was constructed (Figure 2C). The degree sizes of the proteins in the ferroptosis pathway were indicated by the size of the squares, and several proteins were found to be critical for the pathway, including GPX4, FTH1, TP53, SLC11A2, HMOX1, GCLM, STEAP3, SLC11A2, FTL, ATG7, and ATG5. Further, CHRM1, NR3C1, ADRB2, and OPRD1 in the neuroactive ligand-receptor interaction pathway were also found to interact with proteins related to the ferroptosis pathway. Among them, CHRM1 interacted with SLC3A2 and FTH1, NR3C1 interacted with TP53, ADRB2 interacted with PCBP2, and OPRD1 interacted with PCBP2. Therefore, CHRM1, NR3C1, ADRB2, OPRD1, FTH1, TP53, and PCBP2 were defined as the key proteins of the EUO-TT drug pair mediating ferroptosis through the neuroactive ligand-receptor interaction pathway.

### Active Ingredient-Pathway Target Network Construction and Analysis

**Figure 3:**
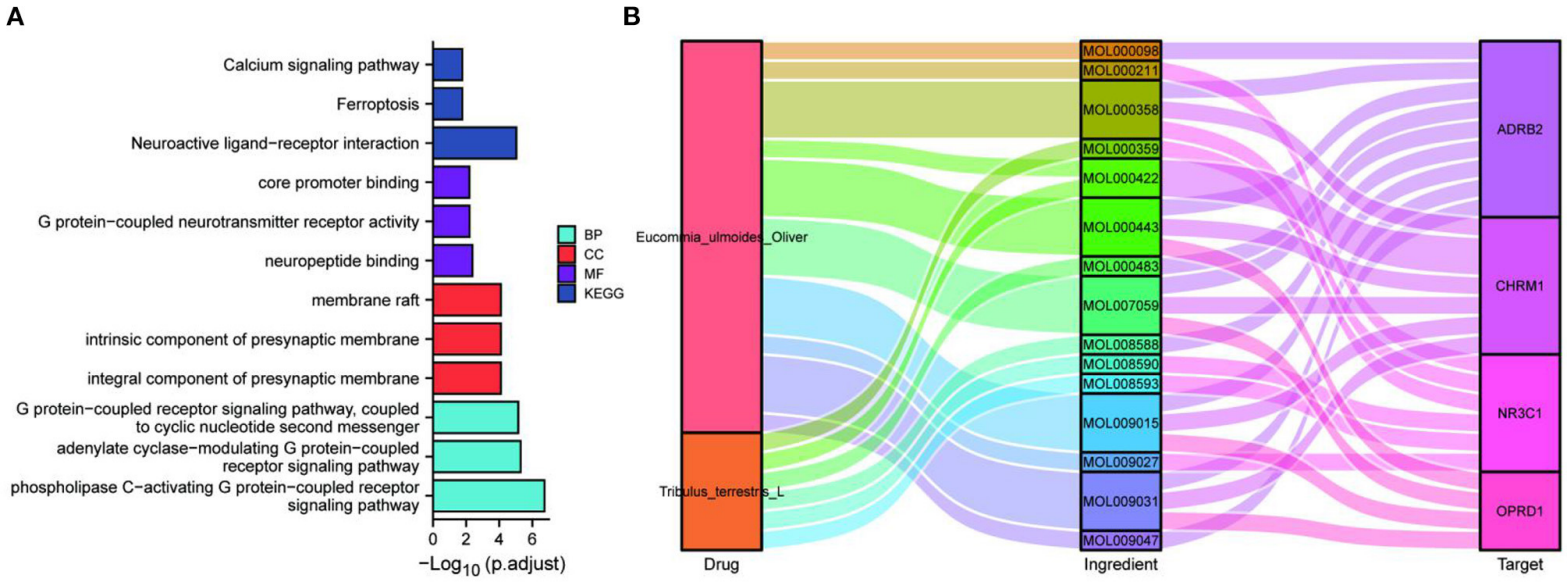
Network construction and pathway analysis of the active ingredient-pathway targets. GO and KEGG enrichment analysis of the key proteins CHRM1, NR3C1, ADRB2, OPRD1, FTH1, TP53, and PCBP2 **(A)**; Sankey plots were used to demonstrate the drug-active ingredients-key neuroactive ligand-receptor interaction pathway targets regulatory network **(B)**.

The key proteins of the drug pair mediating ferroptosis were further used for enrichment analysis. The top-ranked BP, CC, and MF included: core promoter binding, G protein-coupled neurotransmitter receptor activity, neuropeptide binding, membrane raft, intrinsic component of the presynaptic membrane, integral component of the presynaptic membrane, G protein-coupled receptor signaling pathway coupled to cyclic nucleotide second messenger, adenylate cyclase-modulating G protein-coupled receptor signaling pathway, and phospholipase C-activating G protein-coupled receptor signaling pathway. The top-ranked KEGG included: Calcium signaling pathway, Ferroptosis, and Neuroactive ligand-receptor interaction (Figure 3A). Hence, the key proteins may be associated with cell membrane channels, cell signaling, ferroptosis, and the G-protein pathway. Further, Sankey plots were used to illustrate the regulatory network of drug-active ingredient-key targets of neuroactive ligand-receptor interaction pathway (Figure 3B). The “four target proteins with the highest values” refers to the neuroactive ligand-receptor interaction with the four most closely associated proteins in the ferroptosis pathway in the PPI network. Those four key target proteins of the neuroactive ligand-receptor interaction pathway may be regulated by 15 active ingredients of the EUO-TT drug pair.

### Molecular Docking Verification Experiment

**Figure 4:**
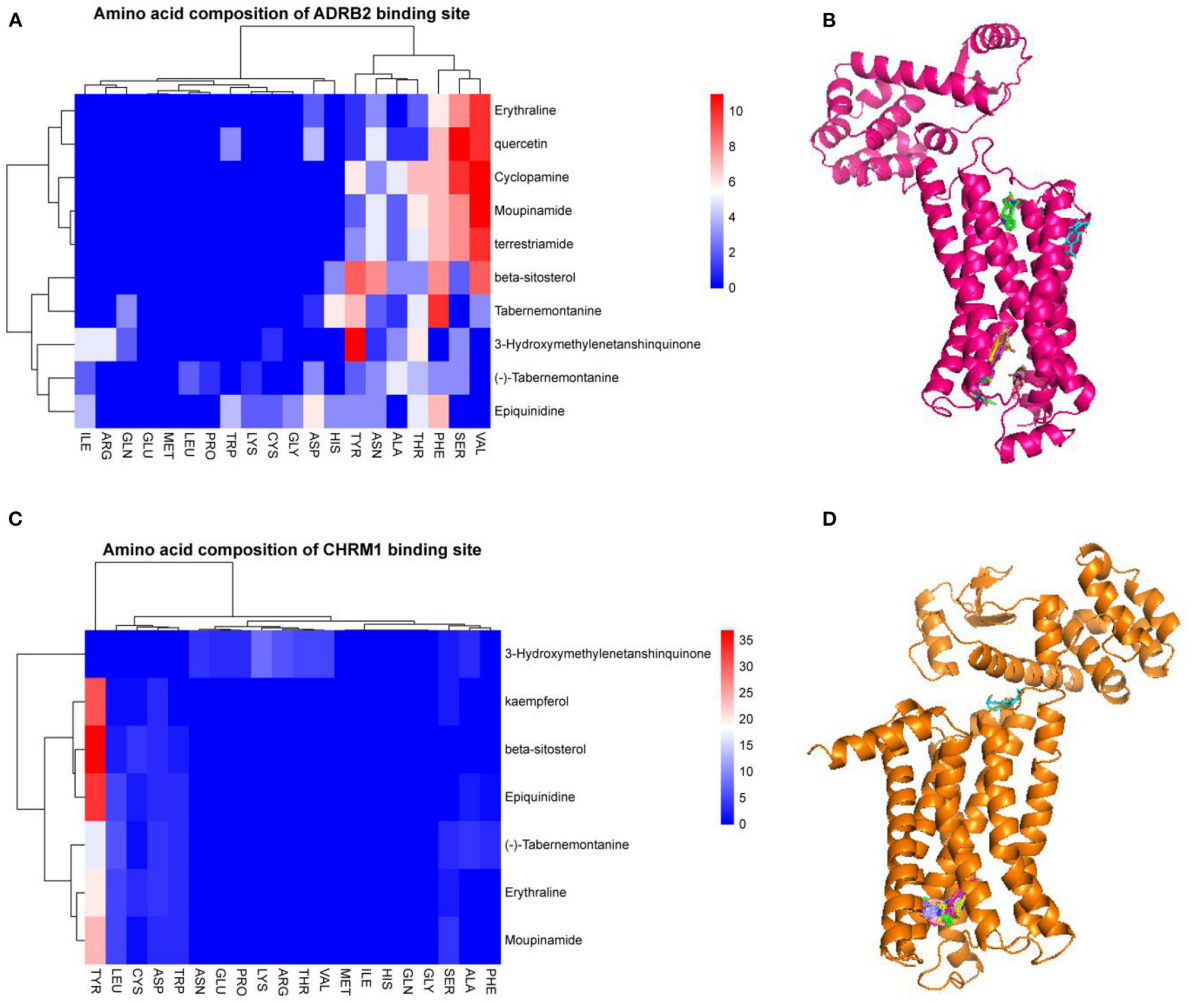
Visualization of molecular docking validation of ADRB2 and CHRM1. Amino acid composition of ADRB2 binding site **(A)**; Bulk molecular docking map of ADRB2 **(B)**; Amino acid composition of CHRM1 binding site **(C)**; Bulk molecular docking map of CHRM1 **(D)**.

**Figure 5:**
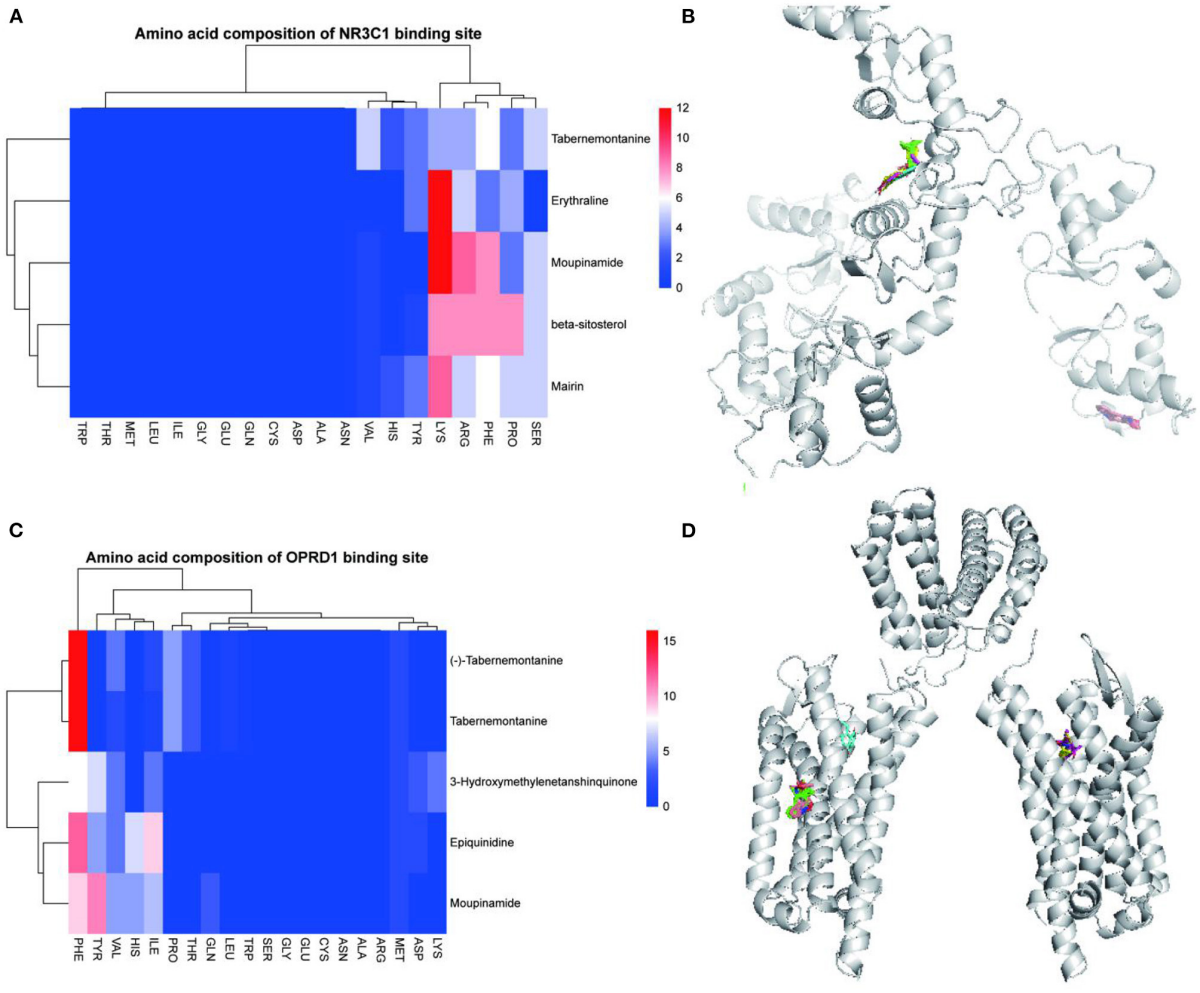
Visualization of NR3C1 and OPRD1 molecular docking validation. Amino acid composition of NR3C1 binding site **(A)**; Bulk molecular docking map of NR3C1 **(B);** Amino acid composition of OPRD1 binding site **(C)**; Bulk molecular docking map of OPRD1 **(D)**.

The 15 active ingredients and four target proteins with the highest values were screened according to the “drug-component-pathway-target” network, and molecular docking experiments were performed. Ultimately, 12 active ingredients were validated for potential target binding to these four proteins (Table 2). The results showed that the binding energy of docking of all proteins and the active ingredients were less than −5 kJ/mol, which indicates a tight binding between the ligand and receptor. The strongest protein-active ingredient bindings were as follows: ADRB2 and cyclopamine (−17.1 kJ/mol), CHRM1 and beta-sitosterol (−13.7 kJ/mol), and NR3C1 and mairin (−12.3 kJ/mol). OPRD1 bound to tabernemontanine (−9.4 kJ/mol), moupinamide (-8.1 kJ/mol), epiquinidine (−9 kJ/mol), 3-Hydroxymethylenetanshinquinone (−9 kJ/mol), and (–)- Tabernemontanine (−9.5 kJ/mol). In the molecular docking validation of ADRB2, CHRM1, NR3C1, and OPRD1 (Figures 4, 5), valine, serine, and phenylalanine were found to be the key amino acids for the action of the active ingredients on ADRB2 (Figure 4A). Erythraline, quercetin, cyclopamine, moupinamide, terrestriamide, and beta-sitosterol had highly similar binding sites on ADRB2 (Figure 4B). Tyrosine was found to be the key amino acid for the action of the active ingredient on CHRM1 (Figure 4C). Kaempferol, beta-sitosterol, epiquinidine, (–)-tabememontanine, erythraline, and mouplinamide had highly similar binding sites on CHRM1 (Figure 4D). Lysine, arginine, and phenylalanine were found to be the key amino acids for the action of the active ingredient on NR3C1 (Figure 5A). Erythraline, moupinamide, beta-sitosterol, and mairin had highly similar binding sites on NR3C1 (Figure 5B). PHE was found to be the key amino acid for the action of the active ingredient on OPRD1 (Figures 5C,D).

**Table 2:** Virtual docking of representative ingredients and proteins.

	**ADRB2**	**CHRM1**	**NR3C1**	**OPRD1**
Terrestriamide	−10			
Tabernemontanine	−9.7		−8.2	−9.4
Quercetin	−10.5			
Moupinamide	−9.9	−9.7	−7.8	−8.1
Mairin			−12.3	
kaempferol		−10		
Erythraline	−12.2	−11.4	−9.4	
Epiquinidine	−9.5	−11		−9
Cyclopamine	−17.1			
beta-sitosterol	−13.2	−13.7	−10.6	
3-Hydroxymethylenetanshinquinone	−8.6	−9.2		−9
(–)-Tabernemontanine	−9.6	−11.3		−9.5

*Binging energy/(kcal/mol)*.

### Construction of IS Prediction Models Based on the Expression Profiles of CHRM1, NR3C1, ADRB2, and OPRD1

**Figure 6:**
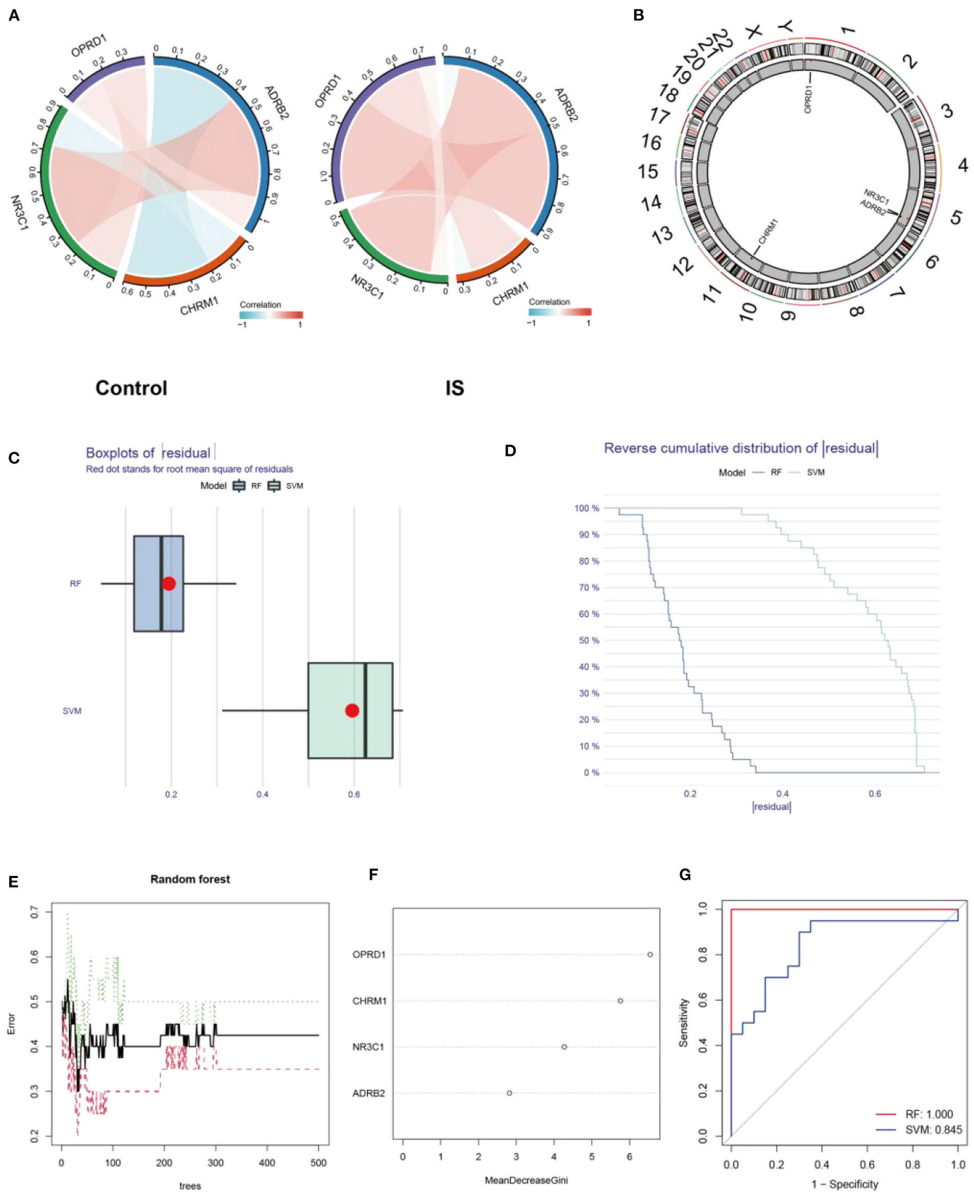
Expression characterization and predictive power analysis of CHRM1, NR3C1, ADRB2, and OPRD1. Correlation analysis of CHRM1, NR3C1, ADRB2, and OPRD1 in the GSE22255 dataset in the control and IS groups, with red indicating positive correlation and blue indicating negative correlation **(A)**; The positions of the four genes CHRM1, NR3C1, ADRB2, and OPRD1 in the chromosome are shown in a circular diagram **(B)**; Boxplot of “residuals” in RF and SVM models **(C)**; Cumulative distribution characteristics of “residuals” in RF and SVM models **(D)**; The variation of error in the random forest model with the number of “trees” included in the model **(E)**; The genes are ranked according to their importance **(F)**; ROC curves reflecting the predictive power of RF and SVM models **(G)**.

Person correlation analysis suggested that in the control group, OPRD1, ADRB2, and NR3C1 were positively correlated with each other, while CHRM1 was found to be negatively correlated with NR3C1 and ADRB2 (Figure 6A). And in the IS group, CHRM1 was found to be significantly and positively correlated with NR3C1 and OPRD1 (Figure 6A). The positions of the four genes CHRM1, NR3C1, ADRB2, and OPRD1 on the chromosomes are shown in a circular diagram (Figure 6B). To evaluate the predictive power of CHRM1, NR3C1, ADRB2, and OPRD1 for IS, RF and SVM models were constructed separately. The boxplot and cumulative distribution characteristics of the “residuals” indicate that the RF model has a lower residual distribution than the SVM model (Figures 6C,D). The variation of error in the random forest model with the number of “trees” included in the model is shown in Figure 6E. Based on the RF model, the importance of each feature was ranked from the largest to the smallest as OPRD1, CHRM1, NR3C1, and ADRB2 (Figure 6F).

The ROC curve analysis revealed that the AUC value of the RF model (AUC = 1.0) was higher than that of the SVM model (AUC = 0.845) (Figure 6G).

### The Construction of a Nomogram Prediction Model, as Well as Its Risk Enrichment Characteristics

**Figure 7:**
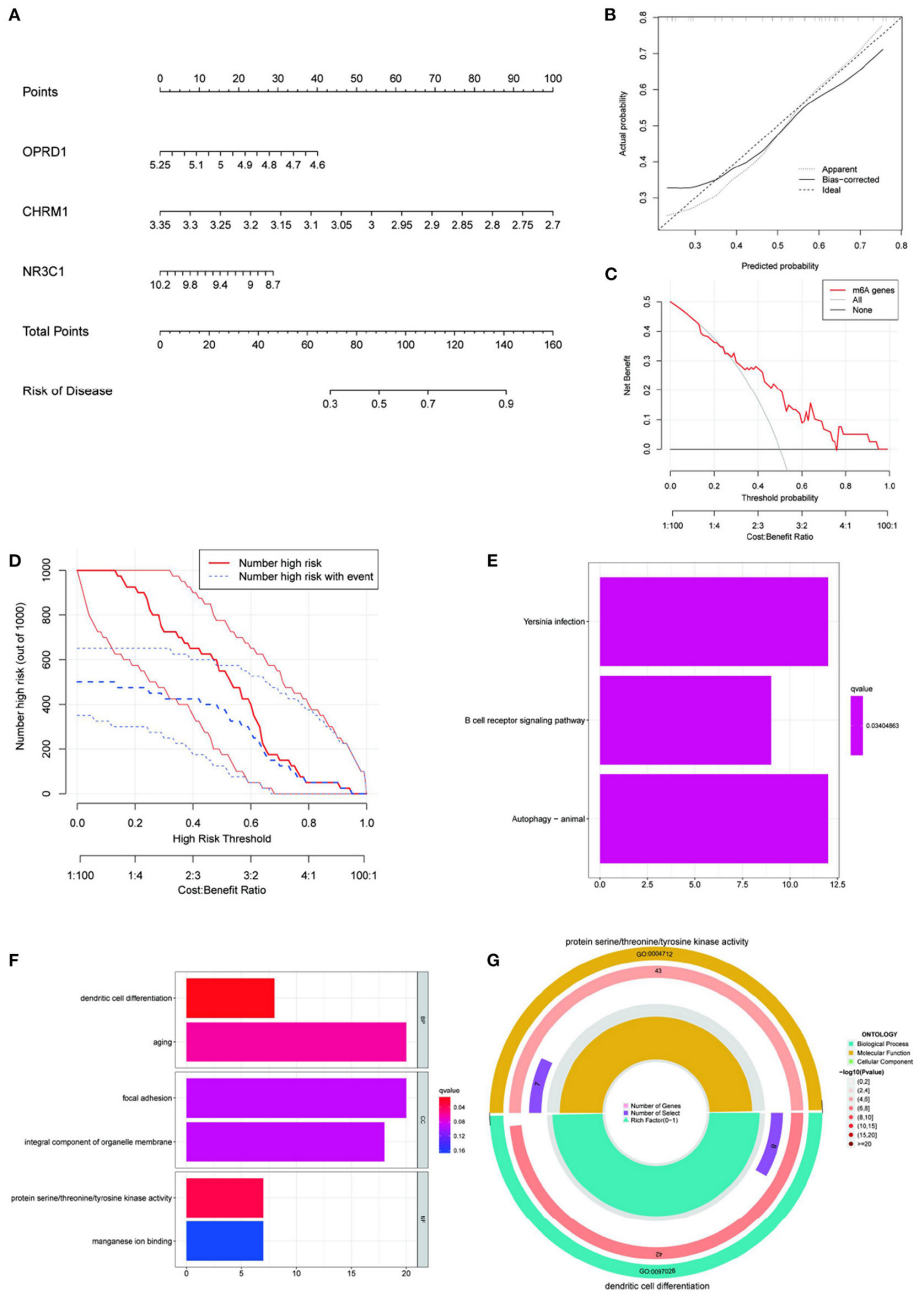
Construction of nomogram prediction model and enrichment analysis based on this model. Construction an IS nomogram model based on the selected genes CHRM1, NR3C1, ADRB2, and OPRD1 **(A)**; Calibration curve showing the diagnostic power of the nomogram model **(B)**; DCA shows that the nomogram model has a good clinical application **(C)**; Clinical impact curves show high diagnostic power of the nomogram model **(D)**; KEGG **(E)** and GO **(F)** enrichment analysis of the genes included in this prediction model **(E,F)**; The most significant GO enrichment pathway associated with this predictive model **(G)**.

To create a visual model for IS prediction, we created a nomogram (Figure 7A) using the four identified genes (CHRM1, NR3C1, ADRB2, and OPRD1). According to the calibration curve, the IS positivity rate predicted by this nomogram was generally consistent with the actual positivity rate (Figure 7B). The DCA and clinical impact curves show that the nomogram model has good clinical application and diagnostic ability (Figures 7C,D). Based on the IS prediction model, all samples in the GSE22255 dataset were scored and divided into high-risk and low-risk groups based on their scores. Differential analysis was performed between the high-risk and low-risk groups. KEGG and GO enrichment were then performed and KEGG enrichment analysis revealed that yersinia infection, B cell receptor signaling pathway and autophagy (animal) were significantly associated with the risk score of this nomogram (Figure 7E). GO enrichment analysis revealed that the risk score of this nomogram was significantly associated with the enrichment of dendritic cell differentiation, aging, focal adhesion, overall composition of organelle membranes, protein serine/threonine/tyrosine kinase activity and manganese ion binding pathways (Figure 7F). Among them, dendritic cell differentiation and protein serine/threonine/tyrosine kinase activity and manganese ion binding were the most enriched (Figure 7G). Therefore, CHRM1, NR3C1, ADRB2, and OPRD1, the target genes of the four EUO-TT drug pair active ingredients, are considered to be risk markers for IS.

## Discussion

This study unraveled the mechanism of action of EUO-TT drug pair in the treatment of hypertension related endothelial injury through the ferroptosis pathway, providing a pharmacological basis for the clinical treatment of hypertension related cerebrovascular endothelial injury using Chinese medicine. CHRM1, NR3C1, ADRB2, and OPRD1, the target genes of the four active ingredients of the EUO-TT drug pair, were considered as risk markers for IS and a visual IS nomogram prediction model was constructed.

Network pharmacology was used to screen the active compounds of EUO-TT L. drug pair and found that the target genes were mainly enriched in the neuroactive ligand-receptor interaction pathway in this research. By constructing the PPI network of neuroactive ligand-receptor interaction pathway-ferroptosis, we found that CHRM1, NR3C1, ADRB2, OPRD1, FTH1, TP53, and PCBP2 were the main target genes of the drug pair. Through the regulatory network of the drug's active ingredients and their key targets in the neuroactive ligand-receptor interaction pathway, we found 15 active ingredients of the drug pair that were involved in the regulation of the key target proteins. Further, molecular docking experiments verified that 12 active ingredients of the drug pair were tightly bound to the target proteins. The expression profiles of CHRM1, NR3C1, ADRB2, and OPRD1 were used to construct IS prediction models. The residual distribution of the RF model was found to be lower than that of the SVM model, and the ROC curve suggested a higher prediction accuracy of the RF model. The RF model rated the genes in ascending order of importance: OPRD1, CHRM1, NR3C1, and ADRB2. Following that, the nomogram prediction model was constructed using the four selected genes (CHRM1, NR3C1, ADRB2, and OPRD1). However, only CHRM1, NR3C1, and OPRD1 were eventually included in the prediction model, as inclusion of these three components was sufficient to reliably predict prognosis in the logistic model. The calibration curve, DCA, and clinical effect curves all indicated that this nomogram model is clinically applicable and diagnostically capable. The enrichment of dendritic cell differentiation and protein serine/threonine/tyrosine kinase activity and manganese ion binding pathways was found to be the most correlated with the prediction results based on nomogram risk scores.

SLC3A2, FTH1, TP53, and PCBP2 are directly or indirectly associated with ferroptosis. SLC3A2 is a SystemXc- heavy chain subunit involved in the maintenance of a redox steady state (56, 57). When SystemXc- is blocked, glutamate and cystine cannot be exchanged, resulting in intracellular glutamate accumulation, reduced glutathione synthesis, and reduced GPX4 activity, which ultimately leads to ferroptosis (58). Further, the inhibition of SLC3A2, a ferroptosis suppressor gene, promotes ferroptosis in tumor and normal cells (57, 59), and hence, its upregulation is associated with poor prognosis of cancer (60). FTH1 (Ferritin heavy polypeptide 1) is an important component of ferritin. Ferritin degradation increases intracellular free iron content and can activate ferroptosis (61–63), while the overexpression of FTH1 can inhibit ferroptosis by impairing ferritinophagy (64). For instance, in the head and neck squamous cell carcinoma, FTH1 suppressed ferroptosis and led to the poor prognosis of the carcinoma (65). Further, FTH1 expression is downregulated in cells sensitive to ferroptosis relative to cells resistant to ferroptosis (66). p53 is an oncogene that regulates the cell cycle, induces apoptosis, and promotes DNA metabolism (67, 68). P53 may also regulate ferroptosis (69–72). Jiang et al. found that P53 inhibited SystemXc- activity and downregulated the expression of solute carrier family 7 member 11, which in turn induced cellular ferroptosis (70). P53 can also catalyse glutamate production by enhancing glutaminase 2 activity, thereby reducing the entry of cystine into cells, reducing glutathione synthesis, and inducing ferroptosis (73). In addition, P53 promotes cellular unsaturated fatty acid oxidation by enhancing the activation of spermidine/spermine N1-acetyltransferase 1, which also leads to cellular ferroptosis (71). Thus, P53 is closely related to ferroptosis. PCBP2 is a multifunctional adaptor protein of the Poly-binding family which is involved in iron metabolism and is an iron chaperone of ferritin (74). However, the specific binding sites of iron and ferritin on PCBP2 have not yet been identified (75). Iron and ROS are promoters and mediators of ferroptosis (76). Iron metabolic homeostasis and lipid peroxidation are crucial for the occurrence of cellular ferroptosis (77). A study found that PCBP2 binding to severely oxidized RNA inhibited apoptosis induced by oxidative stress (78). Moreover, PCBP2 is one of the ferroptosis risk signature genes and can predict the prognosis of adrenocortical carcinoma in combination with the other five ferroptosis risk signature genes (79).

Similar to previous studies, we found that SLC3A2, FTH1, TP53, and PCBP2 were the ferroptosis pathway-associated proteins that interacted with CHRM1, NR3C1, ADRB2, and OPRD1. The NR3C1 protein regulates the genetic features involved in the P53 signaling pathway (80). Further, ADRB2 increases the protein levels of c-myc in pancreatic ductal adenocarcinoma cells through the recruitment of PCBP2 (81). Similarly, in this study, we found that NR3C1 interacted with TP53 andADRB2 interacted with PCBP2. In addition, this is the first study to show that CHRM1 interacted with SLC3A2 and FTH1, and OPRD1 interacted with PCBP2. These results indicated that CHRM1, NR3C1, ADRB2, and OPRD1 may influence the ferroptosis pathway by interacting with the key proteins related to ferroptosis. CHRM1, NR3C1, ADRB2, and OPRD1 were the target proteins related to the neuroactive ligand-receptor interaction pathway. Therefore, for the first time, we show that the neuroactive ligand-receptor interaction pathway may influence the process of ferroptosis through the interaction of its related proteins CHRM1, NR3C1, ADRB2, and OPRD1 with the key proteins of ferroptosis. We further showed that ADRB2 bound most tightly to cyclopamine; CHRM1 to beta-sitosterol; NR3C1 to mairin; OPRD1 to tabernemontanine, moupinamide, epiquinidine; and OPRD1 to tabernemontanine, moupinamide, epiquinidine, 3-Hydroxymethylenetanshinquinone, and (-)-Tabernemontanine. Beta-sitosterol has anti-inflammatory, anti-tumor, antioxidant, anti-hyperlipidemic, and antihypertensive effects (82–84), and moupinamide is also anti-inflammatory (85). Inflammatory processes are involved in the development of hypertension (86, 87). Therefore, it can be hypothesized that moupinamide has potential anti-hypertensive effects. Tanshinone was also found to have a protective effect against cardiac hypertrophy in SHRs (88). Tanshinone slowed the metabolism of the antihypertensive drug cloxacin by inhibiting CYP3A4/CYP2C9 activity (89). Further, the regulation of KV current by tanshinone reduced hypoxic pulmonary hypertension and inhibited hypoxia-induced pulmonary artery wall remodeling (90).

In this study, CHRM1, NR3C1, ADRB2, and OPRD1, the target genes of the four EUO-TT L. drug pair active ingredients, were considered as risk markers for IS. CHRM1 was found to be associated with muscarinic receptor-mediated anti-inflammatory mechanisms (91). Following IS, there is a decrease in B lymphocyte production, which may be related with aberrant NR3C1 expression (92). ADRB2, an apoptosis-related gene, was found to enhance the activity of BDNF/TrkB and cAMP/PKA signaling pathways leading to cellular ischemic injury (93). In addition, polymorphisms in the ADRB2 gene have been found to potentially contribute to a high risk of stroke (94). Common variants of OPRD1 have also been found to be associated with neurodegenerative diseases (95).

In the present study, the active ingredients of EUO-TT L., namely cyclopamine, beta-sitosterol, mairin, tabernemontanine, moupinamide, epiquinidine, and 3-Hydroxymethylenetanshinquinone, were found for the first time to target and bind neuroactive ligand-receptor interaction pathway-related proteins, which in turn regulated the process of cellular ferroptosis and ultimately affected the onset and progression of hypertension. There is a lack of previous studies confirming the association of the alkaloids tabernemontanine and epiquinidine, and the terpenoids cyclopamine, and mairin of EUO with hypertension, ferroptosis, and the neuroactive ligand-receptor interaction pathway. Hence, these pathways can be a new therapeutic target for the prevention and treatment of hypertension.

However, this study also has its shortcomings. The network pharmacology is based on the bioinformatics analysis of the database of herbal targets and disease genes, and thus lacks experimental validation. Hence, further experimental validation is needed to explore the mechanism of action of the drugs or their active ingredients. In addition, this study did not consider the selection of dosage in drug combination and the possible chemical reactions and changes of active ingredients in the process of concoction and decoction of herbal medicines. Furthermore, we also acknowledge that the current research in TCM for the treatment of endothelial injury in hypertension-related cerebrovascular disease is still limited, and therefore we suggest to provide more confirmation and discussion in the future.

## Conclusions

The active ingredients of EUO-TT L. drug pair can act on proteins related to the neuroactive ligand-receptor interaction pathway, which may affect the process of cellular ferroptosis and potentially affect the progression of hypertension. CHRM1, NR3C1, ADRB2, and OPRD1, the target genes of the four EUO-TT L. drug pair active ingredients, were considered as risk markers for IS.

## Data Availability Statement

The original contributions presented in the study are included in the article/supplementary material, further inquiries can be directed to the corresponding author.

## Author Contributions

QZ: software, validation, formal analysis, data curation, and writing-original draft. JY: conceptualization, methodology, supervision, funding acquisition, and writing-review and editing. CY: methodology, conceptualization, project administration, and funding acquisition. XY: data curation and writing-review and editing. YC: software and data curation. All authors contributed to the article and approved the submitted version.

## Funding

This work was supported by the National Natural Science Foundation of China (No. 81804061), Shandong Province Taishan Scholar Construction Project Funds No. 2018-35, and Ji'nan Science and Technology Project (No. 201805078).

## Conflict of Interest

The authors declare that the research was conducted in the absence of any commercial or financial relationships that could be construed as a potential conflict of interest.

## Publisher's Note

All claims expressed in this article are solely those of the authors and do not necessarily represent those of their affiliated organizations, or those of the publisher, the editors and the reviewers. Any product that may be evaluated in this article, or claim that may be made by its manufacturer, is not guaranteed or endorsed by the publisher.

## Abbreviations

GPX4: glutathione peroxidase 4
FTH1: ferritin heavy polypeptide 1
PPI: protein-protein interaction
SHR: spontaneously hypertensive rats
TCMSP: traditional Chinese medicine systems pharmacology database and analysis platform
PPI: protein protein interaction
GO: gene ontology
KEGG: Kyoto encyclopedia of genes and genomes
OB: oral bioavailability
DL: drug-likeness.
